# Zigzag HgTe Nanowires Modify the Electron–Phonon Interaction in Chirality-Refined Single-Walled Carbon Nanotubes

**DOI:** 10.1021/acsnano.2c01647

**Published:** 2022-04-07

**Authors:** Ziyi Hu, Ben Breeze, Reza J. Kashtiban, Jeremy Sloan, James Lloyd-Hughes

**Affiliations:** Department of Physics, University of Warwick, Gibbet Hill Road, Coventry, CV4 7AL, United Kingdom

**Keywords:** single-walled carbon nanotubes, mercury telluride, ultrafast spectroscopy, exciton dynamics, Auger recombination

## Abstract

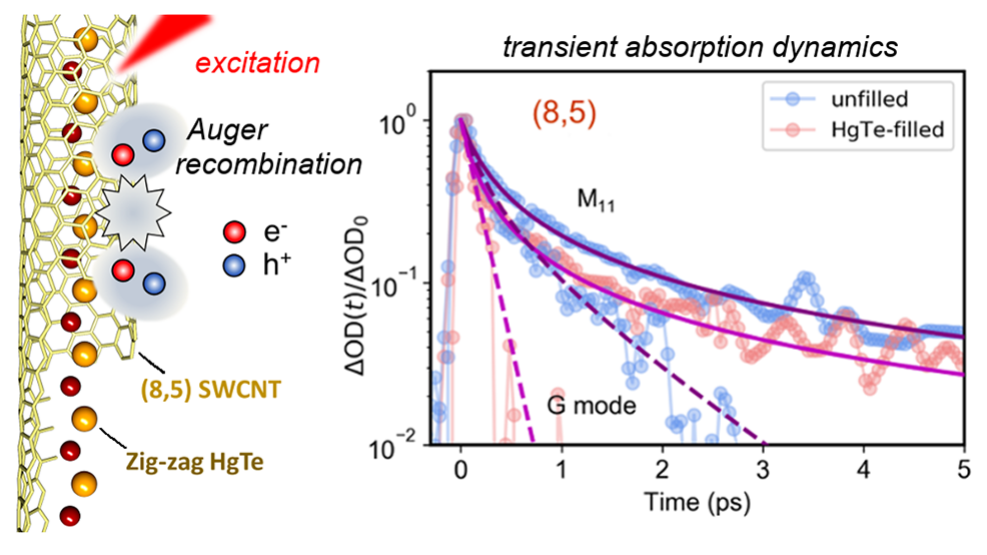

Atomically thin nanowires (NWs) can be synthesized inside single-walled carbon nanotubes (SWCNTs) and feature unique crystal structures. Here we show that HgTe nanowires formed inside small-diameter (<1 nm) SWCNTs can advantageously alter the optical and electronic properties of the SWCNTs. Metallic purification of the filled SWCNTs was achieved by a gel column chromatography method, leading to an efficient extraction of the semiconducting and metallic portions with known chiralities. Electron microscopic imaging revealed that zigzag HgTe chains were the dominant NW geometry in both the semiconducting and metallic species. Equilibrium-state and ultrafast spectroscopy demonstrated that the coupled electron–phonon system was modified by the encapsulated HgTe NWs, in a way that varied with the chirality. For semiconducting SWCNTs with HgTe NWs, Auger relaxation processes were suppressed, leading to enhanced photoluminescence emission. In contrast, HgTe NWs enhanced the Auger relaxation rate of metallic SWCNTs and created faster phonon relaxation, providing experimental evidence that encapsulated atomic chains can suppress hot carrier effects and therefore boost electronic transport.

Carbon nanotubes provide a facile and versatile template for the fabrication of quasi-1D heterostructures. Unique nanoscale materials and composites can be created by various means either internal to the nanotube—such as filling their central void^1^—or by external methods, which include wrapping by conjugated polymers,^2^ selective doping,^3,4^ and overgrowth with van der Waals heterostructures.^5,6^ These methods provide opportunities to tailor the optical and electrical properties of single-walled carbon nanotubes (SWCNTs), as well as creating composites with distinct functionality and where charge or energy can flow between the constituents.^6,7^

Among these various approaches, filling the central pore of a SWCNT with a guest material offers the additional benefit of creating an unconventional 1D nanostructure that cannot be directly synthesized in free space. Previous experimental works have shown that various kinds of materials, including pure metals,^8,9^ metal chalcogenides,^10−14^ metal halides,^15−17^ graphene nanoribbons,^18^ C_60_,^1^ water molecules,^19,20^ white phosphorus,^21^ iodine,^22^ alkane molecules,^23^ and dye molecules,^7^ can successfully be encapsulated inside SWCNTs. When binary compounds crystallize inside narrow SWCNTs, the restricted volume leads to atomically thin nanowires (NWs) that adopt different structures, such as linear chains,^13,17^ zigzag chains,^13^ and helical chains,^17^ depending on the SWCNT diameter.

The extent to which the NW modifies the electronic, excitonic, and vibrational states of the encapsulating SWCNT is of great potential interest as a way to tune the unique physical properties of SWCNTs. While the strong Coulomb interaction in a bare SWCNT leads to large exciton binding energies,^24^ it also leads to rapid scattering processes that involve multiple charge carriers, such as Auger recombination (exciton–exciton annihilation),^25,26^ and which limits the quantum efficiency of light emission.^27,28^ Investigating whether NW encapsulation alters Auger recombination in SWCNTs is therefore of substantial interest. Further, significant electron–phonon coupling can cause nonequilibrium electrons to heat the phonon population if the phonon decay rate is smaller than the electron–phonon scattering rate, as reported for a variety of materials including carbon nanotubes.^29−31^ Electron–phonon coupling is particularly prominent in metallic SWCNT devices under strong electric fields, where electron scattering with a hot optical phonon population limits the tube’s conductivity.^29,30^ Computational models have predicted that phonon–phonon decay rates can be enhanced by encapsulating 1D atomic chains in metallic SWCNTs: the acoustic phonon modes of the encapsulated NWs provide additional routes by which the Γ- and K- point phonons can decay, thereby preventing long-lived phonons and ultimately increasing the current flow achievable along the CNT at high bias.^30^

In this Article we report direct experimental measurements of the influence of nanowire infiltration on electron–phonon coupling and phonon–phonon decay processes in SWCNTs. We focused on HgTe nanowires encapsulated inside narrow-diameter SWCNTs. The choice of SWCNT diameters below 1 nm conveniently allowed optical studies of the excitonic transitions of the CNTs in the technologically relevant UV, visible, and near-infrared ranges. We chose to investigate filling using HgTe NWs, as high filling fractions can be achieved *via* melt infiltration.^12^ Previous work on HgTe NWs in wider SWCNTs (between 1.35 and 1.5 nm) demonstrated a tubular form composed of heavily distorted Hg_2_Te_2_ parallelogram bilayers linked by trigonal bonding^10^ and which displayed sharp Raman vibrational features.^12^ In this study, we report the synthesis of zigzag phase HgTe nanowires inside sub-1-nm-thick SWCNTs using melt infiltration, as confirmed by transmission electron microscopic imaging and diffraction. *Via* a detailed optical characterization, utilizing equilibrium-state fluorescence and Raman spectroscopy, and ultrafast transient absorption spectroscopy (TAS), we demonstrate experimentally that HgTe encapsulation produces substantial modifications to the electron–phonon coupling, phonon–phonon decay, and Auger recombination processes active in SWCNTs. Significantly, the impact of HgTe nanowire infiltration depended on the chirality of the encapsulating SWCNT.

## Results and Discussion

### Structure and Morphology

**Figure 1:**
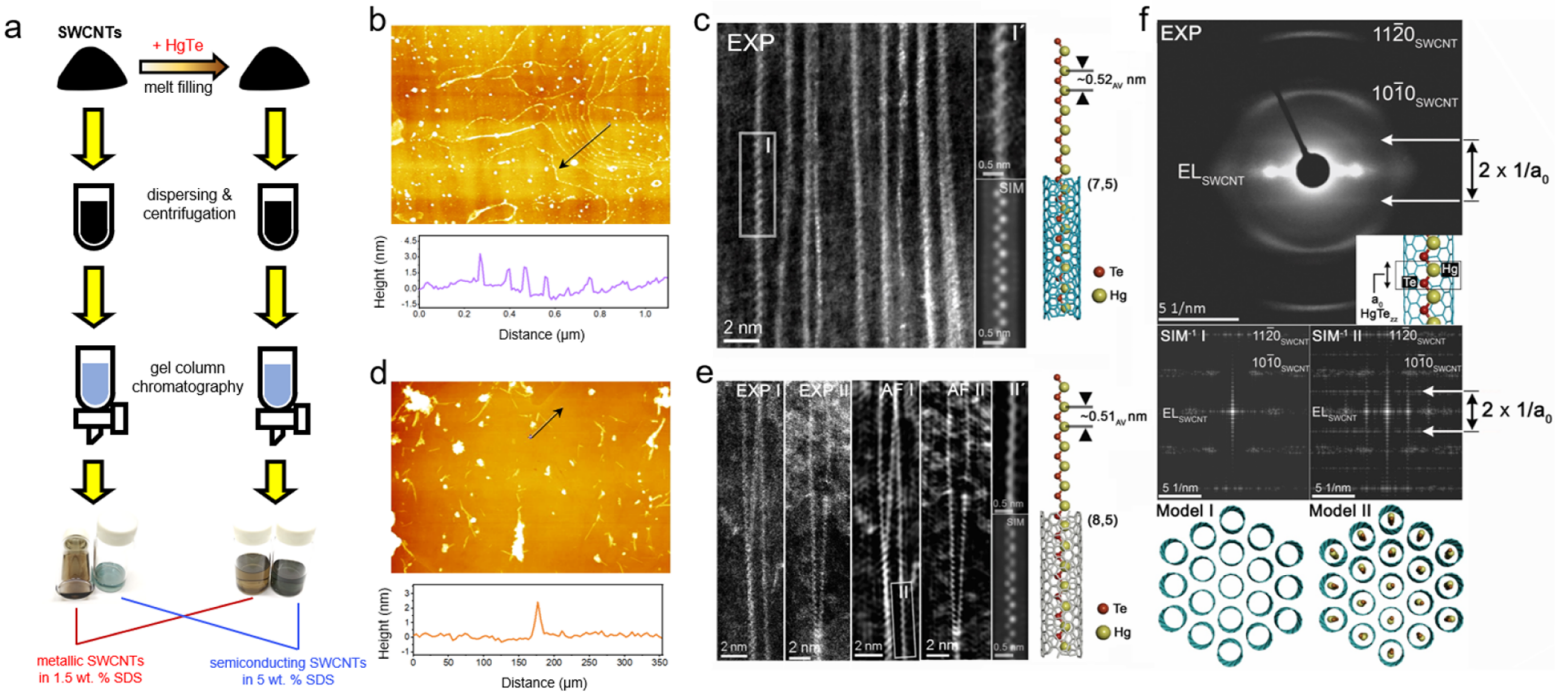
(a) Schematic of SWCNT filling and chirality sorting methodology. (b, d) AFM height and cross-section profiles of the (b) semiconducting and (d) metallic SWCNTs. Arrowed lines illustrate the positions and orientations of the profiles. The width of scanned areas were (b) 5 and (d) 2 μm. (c) ADF-STEM image showing an array of HgTe-filled SWCNTs encapsulated by zigzag chains observed in the semiconducting SWCNT sample. The adapted filtered detail I′ is enlarged from I, and the corresponding simulation (“SIM”) is based on the model, right. (e) As for (c) but two representative regions (EXP I and EXP II). Adaptive filtered versions of these images are shown in AF I and AF II, respectively. A detail from AF I is shown in II and then simulated (“SIM” below) according to the model, right. (f) Experimental ED pattern (top) of a bundle of reagglomerated HgTe-filled semiconducting SWCNTs (inset is the microstructure) with repeating 1D unit cell (lattice parameter *a*_0_). SIM^–1^ I and SIM^–1^ II are FFTs of multislice simulations of models I (unfilled SWCNT bundle) and II (zigzag HgTe-filled bundle), respectively, imaged orthogonally to these end-on projections.

A previous *ab initio* computational study predicted that the formation of zigzag-type SnTe atomic chains is energetically favored for SWCNTs with a size of 0.76–0.95 nm.^13^ We therefore studied SWCNTs within this diameter range (*e*.*g*., (7,5) 0.83 nm, (8,4) 0.84 nm, (10,2) 0.88 nm, (7,6) 0.90 nm, (9,4) 0.92 nm, and (8,6) 0.97 nm) that were filled with HgTe NWs *via* a melt infiltration process. Compared to other larger-diameter (*e*.*g*., over 1 nm) SWCNTs, these sub-1-nm-thick SWCNTs possess larger bandgaps and hence show the advantage of providing discrete near-infrared absorption/photoluminescence features. Along with NW filling inside the SWCNTs, synthesis *via* melt infiltration can also lead to the nanoparticle growth on the outside of nanotubes and introduce guest atoms onto the sp^2^ carbon lattice. Most research on filled SWCNTs thus far has studied nonpurified material or adopted methodologies that can only partially remove the impurities (*e*.*g*., acid washing). Here we adopted a synthesis and refinement process that used ultracentrifugation and gel column chromatography after melt filling to yield semiconducting and metallic species, which presented a green and dark yellow color, respectively, as depicted in Figure 1(a). Annular dark-field scanning transmission electron microscopic (ADF-STEM) images found that a large quantity of nanoparticles coexisted with the SWCNTs after a mild purification treatment (centrifugation under a low force), whereas they were effectively removed under a sufficiently high centrifugal force (Figure S1).

The morphology of the refined, filled SWCNTs was uncovered by the atomic force microscopic (AFM) studies on specimens taken from dilute, dispersed solutions. The semiconducting SWCNT sample was found to consist of nanotubes a few microns long (Figure 1(b)), while the metallic SWCNT sample showed shorter lengths below 500 nm (Figure 1(c)). Besides nanotubes, nanoparticles can also be observed in the AFM images. According to peak force error maps of the same regions, a huge contrast between the nanotubes and nanoparticles was found (Figure S2), suggesting that these particles are likely to be soft organic compounds such as surfactant aggregates, rather than residual catalysts or HgTe nanocrystals.

The detailed atomic structure of HgTe NWs in the chirality-refined semiconducting and metallic SWCNTs was investigated by ADF-STEM (Figure 1(d) and (e)). For both types of samples, discrete zigzag HgTe chains were observed, with an average period of ∼0.52 nm for semiconducting SWCNTs and ∼0.51 nm for metallic SWCNTs, as shown in the representative inset models on the right-hand side of each panel. In Figure 1(f) an electron diffraction (ED) pattern obtained from an aligned bundle of reagglomerated semiconducting SWCNTs filled with zigzag HgTe chains is shown, which contains two extra diffraction features separated by a distance (2 × 1/*a*_0_), where *a*_0_ is the period of a quasi 1D zigzag HgTe chain, as depicted in the model inset into the experimental ED pattern. As can be seen from the two ED simulations in the bottom insets (SIM^–1^ I and SIM^–1^ II), which correspond to the diffraction behavior of model I (empty SWCNTs) and model II (SWCNTs filled with randomly oriented zigzag HgTe chains), the extra diffraction feature arises from the 1D zigzag chains. The value for *a*_0_ measured from the experimental diffraction pattern corresponds to ∼0.525 nm, consistent with measurements obtained from the real-space images in Figure 1(d) and (e) within experimental error. Analysis based on the energy-dispersive X-ray (EDX) spectrum yields a Hg:Te ratio of 1:1, further verifying the formation of HgTe (Figure S3).

### Optical Absorbance: Excitons and Plasmons

**Figure 2:**
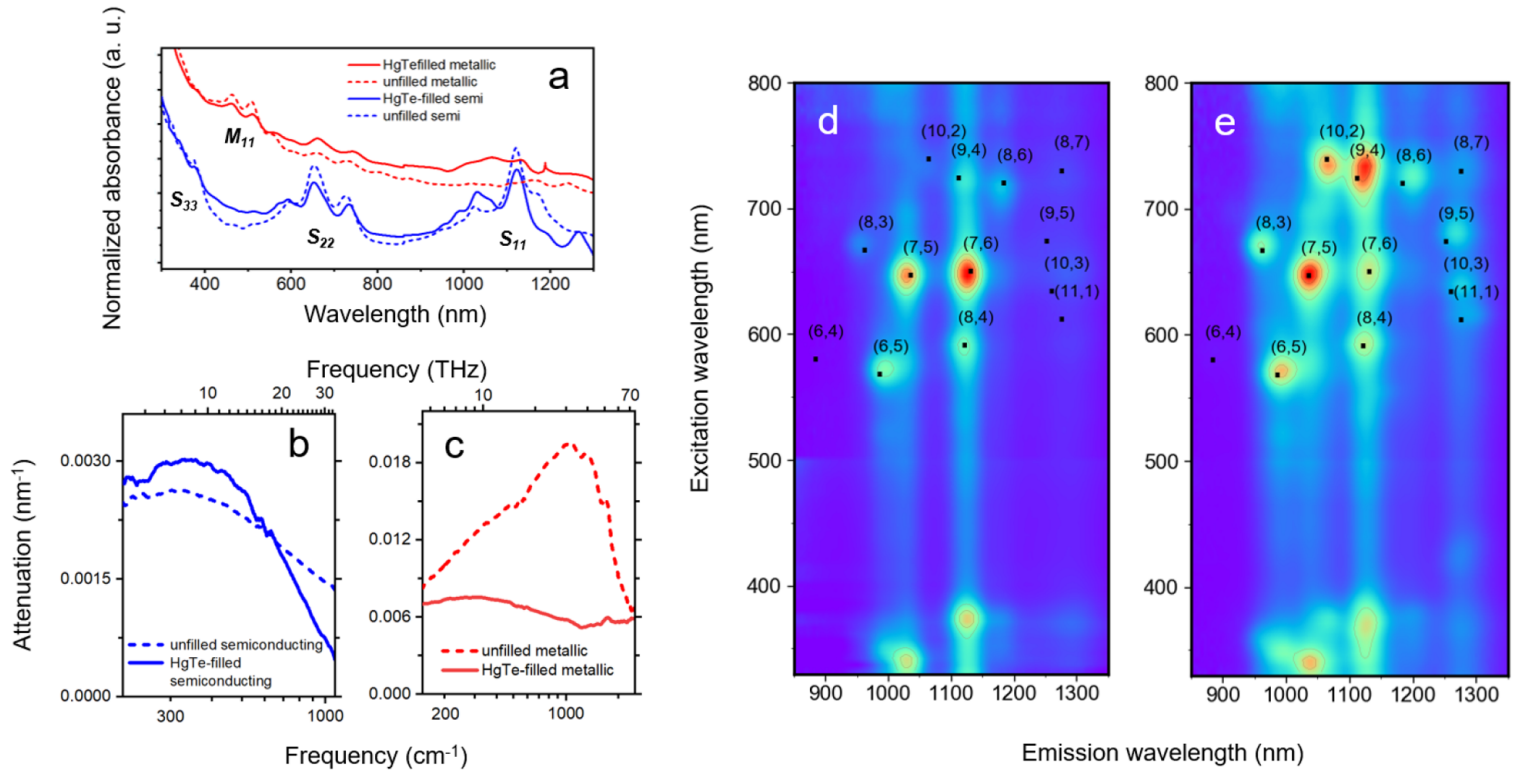
(a) UV–vis–NIR absorption of the unfilled and HgTe-filled SWCNT solutions. The spectra are normalized to the absorbance values at 400 nm, where there are no expected excitonic features. (b, c) Room-temperature infrared attenuation of the semiconducting and metallic SWCNT films divided by their thicknesses. (d, e) PLE maps of the solution-state (d) unfilled and (e) HgTe-filled semiconducting SWCNTs. Black dots show the S_22_ and S_11_ wavelengths for each (*n*,*m*) species according to the empirical model.^32^

We now discuss the optoelectronic properties of the HgTe/SWCNT heterostructures, as assessed by equilibrium-state optical spectroscopy on solutions (Figure 2) and thin films (Figure S4). Spectra were normalized at 400 nm in order to correct for variations in CNT concentration between different solutions. The ultraviolet–visible–near-infrared (UV–vis–NIR) absorbance spectra (Figure 2(a)) reveal several absorption lines corresponding to the different excitonic transitions of SWCNTs (*e*.*g*., S_11_, S_22_, S_33_, and M_11_). For the semiconducting samples, two dominant absorption lines in the regions 645–650 nm and 1120–1130 nm were found, linked to the S_22_ and S_11_ transitions of (7,6), and are suggestive of a dominant (7,6) chirality. For the metallic SWCNT samples, two absorbance lines located at ∼460 and ∼507 nm are evident, which can be ascribed to the M_11_ excitonic transitions. For the thin films, a marked red-shift of the excitonic peaks can be observed in Figure S4 (*e*.*g*., S_22_ and S_11_ bands red-shifted to ∼670 and ∼1190 nm and two M_11_ bands red-shifted to ∼468 and ∼510 nm), which can be attributed to strong rebundling when the nanotubes condensed into films.

To investigate the impact of HgTe filling on the conductivity of free carriers, mid- and far-infrared attenuation spectra of thin films of materials produced by the vacuum filtration method were obtained, as reported in Figure 2(b) and (c). The broad absorption maxima in the free-carrier absorption regime (below 3000 cm^–1^) result from the axial plasmon resonance of finite-length nanotubes.^4,33−35^ The absorbance profiles for semiconducting and metallic SWCNTs are distinctly different. The semiconducting SWCNTs exhibited a narrower absorption peak centered at around 300 cm^–1^, while the metallic tubes had a broader absorption peak around 1000 cm^–1^. We note that the theory of axial plasmons in SWCNTs^33^ predicts the plasmon resonance frequency to vary inversely with tube length, *l*, according to ω_0_ = πv_*q*_(*l*, *d*, *N*)/*l*, where *v*_*q*_(*l*, *d*, *N*) is the plasmon mode velocity, a function of the tube diameter *d* = 0.88 nm and length and also of the number of nanotubes in each bundle.^33^ The bundle dependence has not been previously considered in comparisons between the experimental plasmon frequency and theory.^34−36^ For the thin films studied here, each bundle can contain up to hundreds of SWCNTs. Since ,^33^ the plasmon frequency is higher than would otherwise be expected for these tube lengths (over 1 μm for semiconducting tubes and around 200 nm for the metallic tubes, according to AFM).

After HgTe filling, the infrared attenuation of the semiconducting SWCNTs was found to remain almost the same, while that of the metallic SWCNTs displayed a significant reduction in strength and a substantial red-shift. These results indicate that HgTe filling did not lead to a significant change in the equilibrium charge carrier density of the semiconducting SWCNTs but can efficiently lower the free carrier density in the metallic SWCNTs. Pseudogaps have been reported to open when metallic tubes form bundles,^37^ which might therefore reduce the free carrier density and conductivity of bundles of metallic SWCNTs. In this case however the pronounced difference between reference and filled metallic samples, which had similar bundle number, allows us to infer that the IR absorbance was changed by the presence of HgTe. We advance the hypotheses that the presence of HgTe NWs either modifies the band structure of each individual metallic SWCNT or alters the pseudogap derived from intertube coupling in bundles, but further work is needed to clarify the observed changes.

### Excitonic Photoluminescence

The excitonic absorption and emission energies for different chirality semiconducting SWCNTs can be quantified using photoluminescence excitation (PLE) maps under S_22_ or S_33_ excitation. Following absorption, rapid relaxation to the lowest (S_11_) excitonic state occurs before light is emitted. PLE maps of the unfilled (Figure 2(d)) and HgTe-filled (Figure 2(e)) semiconducting samples are shown, along with the transition wavelengths expected from the empirical Kataura model^32^ for each (*n*,*m*) (black filled squares). The PLE map of the unfilled semiconducting sample reveals that (7,6) and (7,5) are the two most abundant (*n*,*m*) species. After HgTe filling, (8,3), (7,5), (10,2), (9,4), and (8,6) displayed a dramatic relative enhancement in their fluorescence, while the (7,6) fluorescence strength was relatively lower. PLE profiles averaged over the entire spectral window and for windows covering only particular species are shown in Figures S5 and S6. The higher fluorescence intensity from HgTe-filled semiconducting SWCNTs points toward either reduced nonradiative interactions for *S*_11_ excitons or a greater radiative rate. Later in the Article we turn to transient absorption spectroscopy to investigate this further.

By exciting the semiconducting samples at NIR wavelengths (850–1000 nm) close to S_11_, additional PLE signals were resolved (Figure S7, black circles) beneath the first-order Rayleigh scattering line, which can be assigned to emission at the G-mode phonon sidebands of the S_11_ excitons.^38^ After HgTe NW filling the semiconducting SWCNTs presented an additional signal peak centered at ∼1060 nm (Figure S7), further evidencing stronger emission from (10,2) CNTs after filling.

In addition to changes in fluorescence intensity, small shifts in maxima were observed for specific (*n*,*m*) species: for instance the fluorescence peaks of (7,5), (9,4), and (8,6) shifted diagonally toward larger excitation and emission wavelengths. In contrast, the PLE peak for (6,5) SWCNTs (0.75 nm diameter) was not altered, suggesting that filling did not alter the energy of the excitonic states or that the narrowest tubes were not as filled.

### Phonons and Electron–Phonon Coupling Assessed by Raman Spectroscopy

**Figure 3:**
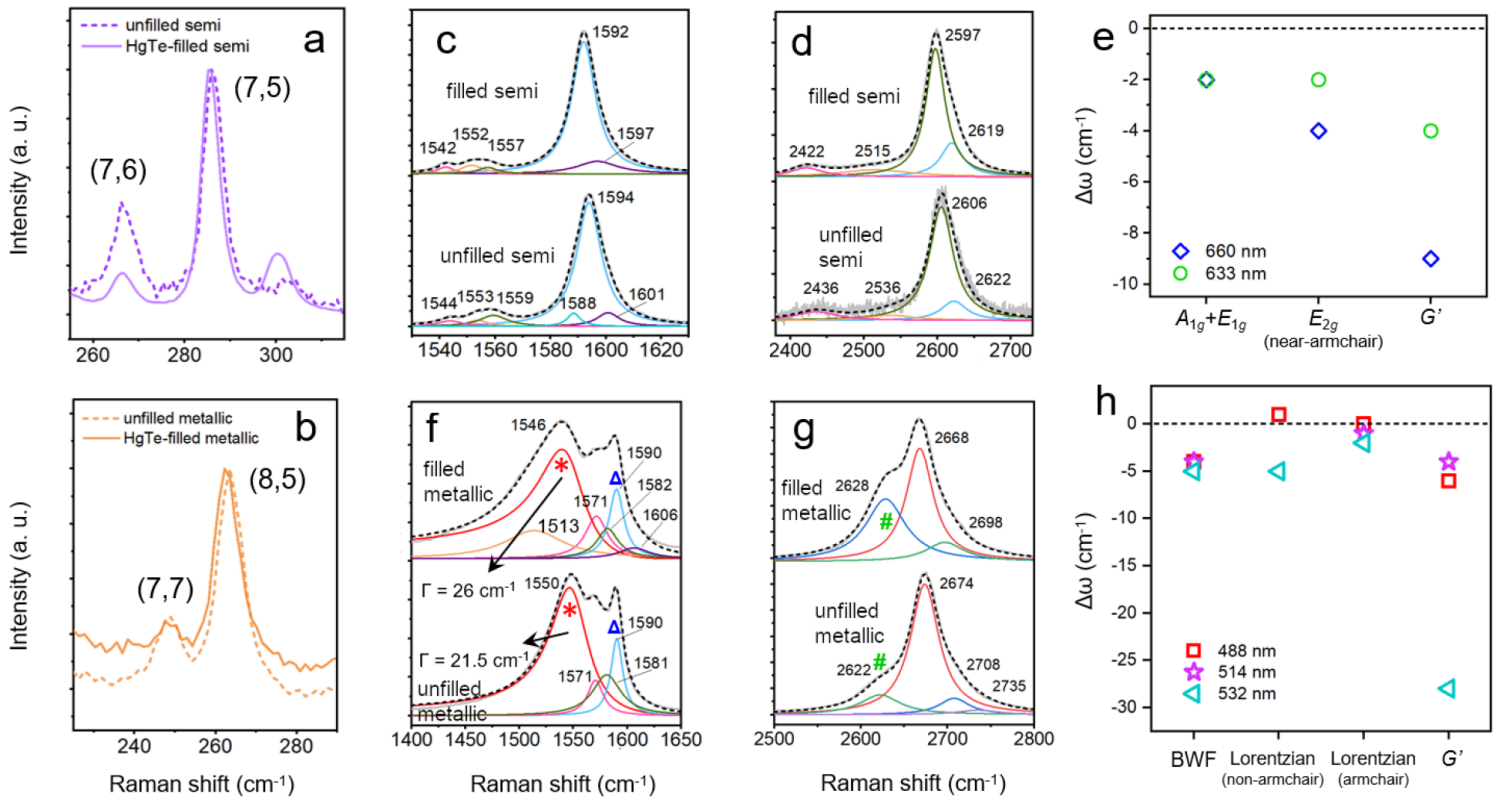
Raman spectra of the RBMs of (a) semiconducting SWCNTs (under 660 nm excitation) and the (b) metallic SWCNTs (488 nm excitation), with (solid lines) and without (dashed lines) HgTe filling. The (c) G and (d) G′ modes of the semiconducting samples are shown under 660 nm excitation, along with (e) the spectral shifts caused by HgTe filling for the strongest modes. The (f) G and (g) G′ modes of the metallic samples are reported under 514 nm excitation. The BWF line of non-armchair metallic species (red asterisks) and an additional Lorentzian component at 1590 cm^–1^ (blue triangles) are also shown. The green number sign marks a second component of the G′ mode of the metallic samples. (h) Spectral shifts created by filling the metallic samples.

The anti-Stokes Raman resonances were investigated in order to confirm chirality assignments and to examine the vibrational modes and electronic properties of the HgTe-filled nanocomposites. Radial breathing modes (RBMs) of the nanotubes were observed at Raman shifts of 200–350 cm^–1^ (Figures 3(a),(b) and S8), in accord with the small (<1 nm) diameters of these SWCNTs. Under an excitation wavelength of 660 nm (an energy of 1.88 eV), two intense peaks were found at 267 and 286 cm^–1^ (Figure 3(a)) for the semiconducting SWCNTs. According to the relation *d*_t_ = *a*/ω_RBM_, where *a* = 237 nm cm^–1^ and ω_RBM_ is the wavenumber of the RBM,^39^ these two RBM features correspond to *d*_t_ = 0.89 nm and *d*_t_ = 0.83 nm, *i*.*e*., (7,6) and (7,5) SWCNTs, respectively. In contrast, for the metallic samples, the RBMs are reported in Figure 3(b) (488 nm excitation, close to the *M*_11_ absorption peaks) and in Figure S8(b1) and (b2) (under excitation at 514 and 532 nm, respectively)^40^ and result from (8,5) tubes (∼266 cm^–1^) and small fractions of (7,7) tubes (∼250 cm^–1^).

It is noted that HgTe NW filling results in a slight softening (or red-shifting) of the RBM Raman shift for both semiconducting and metallic SWCNTs. This is somewhat unanticipated, as molecular filling normally favors the hardening (blue shifting) of the RBM, as previously demonstrated by both theoretical^41^ and experimental^19,23^ results. The reason behind this warrants further study.

The Raman spectra of SWCNTs at higher frequencies, such as the tangential mode (G mode) and the double resonance G′ mode, provide a further route to investigate the changes in the electronic properties of the SWCNTs induced by the HgTe zigzag filling. In the current work, five laser excitation wavelengths were applied (488, 514, 532, 633, and 660 nm, corresponding to 2.54, 2.41, 2.33, 1.96, and 1.88 eV), making it possible to uncover the resonant Raman features of different (*n*,*m*) species in semiconducting and metallic samples. Raman spectra were analyzed by subtracting a linear baseline and then fitted by a superposition of Lorentzian functions.

The Raman-active G band of SWCNTs contains multiple peaks as a result of the curvature of the SWCNT, which yields different C–C vibrational frequencies for the axial and circumferential directions.^42,43^ Spectra of the G modes for the semiconducting SWCNTs under 660 and 633 nm excitation are shown in Figures 3(c) and S9(a). In both cases, the best fit to the experimental spectrum required five or six Lorentzians, which can be attributed to zone-center phonon modes with A_1g_, E_1g_, and E_2g_ symmetries.^44^ The strongest, higher-frequency band (or G^+^ band) consists of two Lorentzians centered at ∼1590 and ∼1600 cm^–1^, which are assigned to the A_1g_+E_1g_ and E_2g_ modes, respectively. The low-frequency band (or G^–^ band) comprised three Lorentzians, which can be assigned to another E_2g_ and two A+E modes. The double-resonance G′ modes are linked to two-phonon scattering processes near the K-point^45,46^ and are reported in Figure 3(d) for the semiconducting SWCNTs (along with Figure S9(b)). One strong peak was observed around 2600 cm^–1^, which was decomposed into Lorentzian peaks.

Filling with HgTe NWs red-shifted all of the G and G′ modes of the semiconducting SWCNTs, as indicated by the difference in Raman peak position after filling, Δω, in Figure 3(e). While the G^+^ resonance position is insensitive to CNT diameter, it red-shifts when relatively more electrons are present on the CNTs,^43,47^ suggesting the filling has acted like a weak donor.

The G and G′ spectra of the metallic filled and unfilled SWCNTs (Figures 3(f,g), S9(c,d), and S10(b)) are more complex than the spectra for the semiconducting samples. The so-called G^–^ mode of metallic tubes, covering the 1500–1550 cm^–1^ region in Figures 3(f) and S9(c), displays a distinct spectral shape that can be described by the Breit–Wigner–Fano (BWF) line shape and is thought to result from the down-shifting and broadening of the axial tangential Raman mode after strong coupling to the electronic continuum of metallic tubes.^48^ While the BWF line can be clearly seen for non-armchair metallic SWCNTs, such as (8,5), it is absent in the Raman spectra of armchair SWCNTs such as (6,6) or (7,7). The asymmetric BWF line for non-armchair metallic SWCNTs can be expressed as^49^
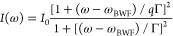
1where the parameter *q* describes the coupling strength between the phonon and the electronic continuum, ω_BWF_ is the wavenumber of the maximum intensity *I*_0_, and Γ describes the line broadening. Here the BWF line at 1540–1550 cm^–1^ and a Lorentzian component at ∼1580 cm^–1^ are assigned to the non-armchair metallic species (8,5), while a narrower Lorentzian peak at ∼1590 cm^–1^ is linked to the G^+^ mode of armchair species^50,51^ or of residual semiconducting tubes.^48^ The G′ modes of the metallic tubes (Figure 3(g)) showed a clear double-peak structure, which is only found for non-armchair metallic tubes^43^ and which results from trigonal warping around the K-point.^43^

After filling with HgTe chains, the BWF feature further downshifts and broadens in width (Figure 3(f)), with a filling-induced shift Δω ≃ −5 cm^–1^ (Figure 3(h)). Under 488 nm excitation the spectral broadening increased from Γ = 24 cm^–1^ to 32 cm^–1^ after filling (from 21.5 cm^–1^ to 26 cm^–1^ with 514 nm excitation). A summary of the fit parameters for the BWF models is provided in Table S1. The line width of this Raman mode was previously linked to the electron–phonon coupling rate *via* electrostatically gated Raman spectroscopy on individual metallic SWCNTs.^52^ We therefore deduce that the HgTe chains induce a stronger electron–phonon interaction in metallic tubes.

The intensity ratio of the disorder band (D band) to the G^+^ band, a value reflecting the degree of imperfection of the sp^2^ carbon lattice,^53^ showed nearly no change after the HgTe filling experiment (Figure S11), indicating that any structural damage caused by the filling process is negligible. This result, in conjunction with the TEM results, provides convincing proof that the evolution of the high-frequency SWCNT Raman modes was caused by HgTe NW filling, rather than as a result of damage to the SWCNTs or extraneous material.

### Summary of Steady-State Optical Properties

The experimental results from optical spectroscopy indicate that the impact of HgTe zigzag NW inclusion depends on the chirality of the encapsulating SWCNTs. Raman spectra indicate that filling enhanced electron–phonon coupling in the metallic tubes, while IR absorbance demonstrated that the long-range conductivity of thin films of metallic SWCNTs was suppressed by filling. For the semiconducting tubes, HgTe NWs did not result in substantial changes in film conductivity, but did subtly shift the excitonic transition energies and did show a small amount of electron transfer from the HgTe NWs to the carbon nanotubes.

### Femtosecond Transient Absorption Spectroscopy

**Figure 4:**
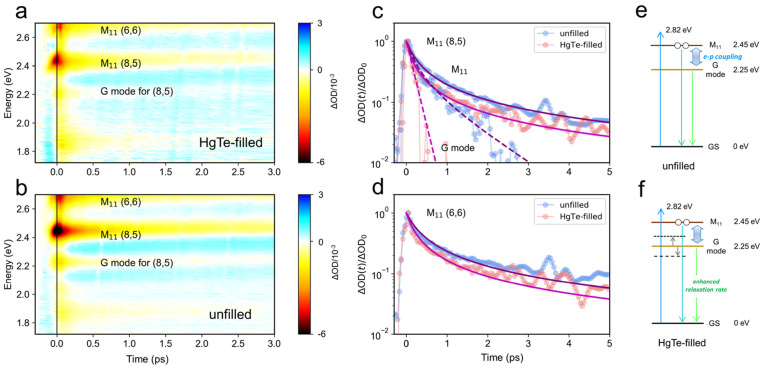
(a, b) Variations of the TA signals (ΔOD) against the pump–probe time delay and the probe energy for the solution-state (a) HgTe-filled and (b) unfilled metallic SWCNTs. The applied pump energy and fluence were 2.82 eV (440 nm) and 1.13 mJ cm^–2^ per pulse, respectively. (c, d) TA kinetics of the M_11_ bands for (8,5) and (6,6) as well as the G mode of (8,5). Fits (purple for the unfilled and magenta for the HgTe-filled) are as described in the text, based on the bimolecular rate equation model in eq 3. (e, f) Energy diagrams and possible relaxation pathways for (8,5) (e) without and (f) with HgTe filling. The arrow denotes electron–phonon (e–p) coupling, and the upward blue arrow marks the pump energy. The extra phonon states (dashed lines) of the infiltrated CNTs provide additional nonradiative relaxation pathways that boost the Auger rate.

To investigate the differing consequences of HgTe NW incorporation for metallic and semiconducting SWCNTs in more detail, we used femtosecond transient absorption (TA) spectroscopy to study dispersed tubes in solution. This allowed the dynamics of excitons on isolated metallic or semiconducting tubes to be resolved. Experimental TA signals (change in absorbance, Δ*A*) against the probe energy and pump–probe delay are shown in Figure 4(a) and (b) for the metallic samples. In the heat maps, several negative photobleach (PB) features can be discerned in the experimental spectral range (dark red areas), which correspond to the M_11_ excitonic states of (6,6) and (8,5) chiralities. The feature at 2.45 eV was assumed to result mainly from (8,5) rather than (7,7), which has similar M_11_ energy, by virtue of the larger RBM weighting of (8,5) (Figure 3(b)). Also evident in the TA heat maps is a PB feature at around 2.25 eV, 0.2 eV below M_11_ for (8,5), which we assign to an exciton–phonon sideband created by the G mode. The strong electron–phonon coupling in SWCNTs can create phonon sidebands that can be seen directly in the absorption spectra of SWCNTs.^54^

In Figure 4(c) and (d) the dynamics of the (8,5) and (6,6) features are reported. The observed nonexponential transient absorption dynamics were modeled by assuming that the normalized transient absorption change ΔOD(*t*)/ΔOD_0_ = *n*(*t*)/*n*_0_, where *n*(*t*) is the population of excitons and *n*_0_ = *n*(*t* = 0), and by assuming a rate of change of *n*(*t*) given by
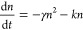
2Here the first term, including the constant γ, describes bimolecular exciton recombination processes such as Auger recombination, while *k* tracks monomolecular decay *via* radiative and nonradiative processes. After normalization by *n*_0_, the time evolution of the transient absorption signal for times *t* > 0 is
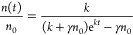
3and hence *k* and γ*n*_0_ are the fit parameters. We adopted this approach to fit our TA data because the maximum exciton density *n*_0_ is hard to quantify precisely. For the M_11_ states of (8,5) and (6,6) tubes, the decay dynamics were bimolecular throughout the experimental time window, which is the case when the monomolecular rate is much smaller than the Auger recombination rate. Good agreement between model and experiment was therefore found without the need for a contribution from *k* (*i*.*e*., *k* ≪ 0.1 THz), by taking γ*n*_0_ = 4.13 ± 0.06 THz and 3.27 ± 0.09 THz for unfilled (8,5) and (6,6) tubes. A full list of parameters for the fitted dynamic curves at different probe energies can be found in Table S2. The experiments were conducted under the same nonresonant excitation conditions and on samples with similar absorbance. Assuming the same internal conversion rate to M_11_ excitons for each sample (during the first 100 fs time resolution of the pump–probe experiment), we therefore assume *n*_0_ did not vary with chirality or filling and instead discuss the mechanisms that can alter γ.

The lower Auger rate γ for unfilled (6,6) SWCNTs in comparison to (8,5) can be understood as follows. Within a parabolic two-band approximation, *i*.*e*., ignoring any electron-scattering processes between subbands and neglecting the electron–phonon interaction, the Auger rate for nanotubes can be estimated from perturbation theory to scale as γ ∝ (*E*_b_/*E*_g_)^3^, where *E*_b_ is the exciton binding energy and *E*_g_ is the single-particle energy gap.^26^ The narrower diameter of (6,6) tubes (0.814 nm) increases *E*_g_ (as well as M_11_) relative to that of (8,5) tubes (0.889 nm diameter). Hence the lower γ for (6,6) tubes may result solely from diameter-driven changes to the electronic band structure. However, this discussion ignores the important role that electron–phonon coupling plays in metallic carbon nanotubes, as evident from their Raman features (discussed above) and also evidenced by time-domain atomistic simulations of Auger decay, where two-particle exciton–exciton Auger decay processes were found to involve phonon-assisted transitions at energies up to and including the G mode.^55^ Hence changes to the G mode’s strength, energy, and line width may be expected to alter Auger decay rates.

HgTe zigzag filling directly increased the extracted Auger rates to γ*n*_0_ = 7.17 ± 0.13 THz and 5.07 ± 0.20 THz for (8,5) and (6,6) metallic SWCNTs. As there was negligible change in the M_11_ energy after filling, the around 60% higher γ for the filled tubes can be interpreted as resulting from stronger electron–phonon coupling, leading to a greater Auger rate. Further evidence for the enhanced role of electron–phonon coupling comes from the G-mode sideband (at 2.25 eV), the dynamics of which are reported in Figure 4(c). Exciton–exciton annihilation creates a single, higher energy electron–hole pair, which then relaxes by optical phonon emission. The G-sideband’s dynamics for the unfilled (8,5) tubes was modeled (dashed blue line) by the same Auger term (γ*n*_0_ = 4.13 THz) but with an additional monomolecular channel at rate *k* = 1.0 THz, corresponding to a lifetime τ = 1/*k* = 1.0 ps. This is in excellent agreement with the lifetime of the G-mode phonon, τ = 1.1 ± 0.2 ps, measured directly by time-resolved Raman spectroscopy.^54^ After HgTe zigzag filling, however, the G-mode sideband decayed much more rapidly and was fit (dashed red line) by a single-exponential decay with lifetime τ = 0.16 ps. This faster decay may signify that HgTe filling strains the CNTs,^17^ thereby boosting the anharmonic relaxation pathways for the G-mode phonons to relax into other phonons within the SWCNT. Alternatively, the presence of encapsulated insulating nanowire chains has been shown theoretically to increase hot phonon relaxation rates by providing extra phonon decay channels.^30^ The acceleration of the Auger relaxation rate in (8,5) SWCNTs due to HgTe NW filling is shown pictorially in Figure 4(e) and (f).

**Figure 5:**
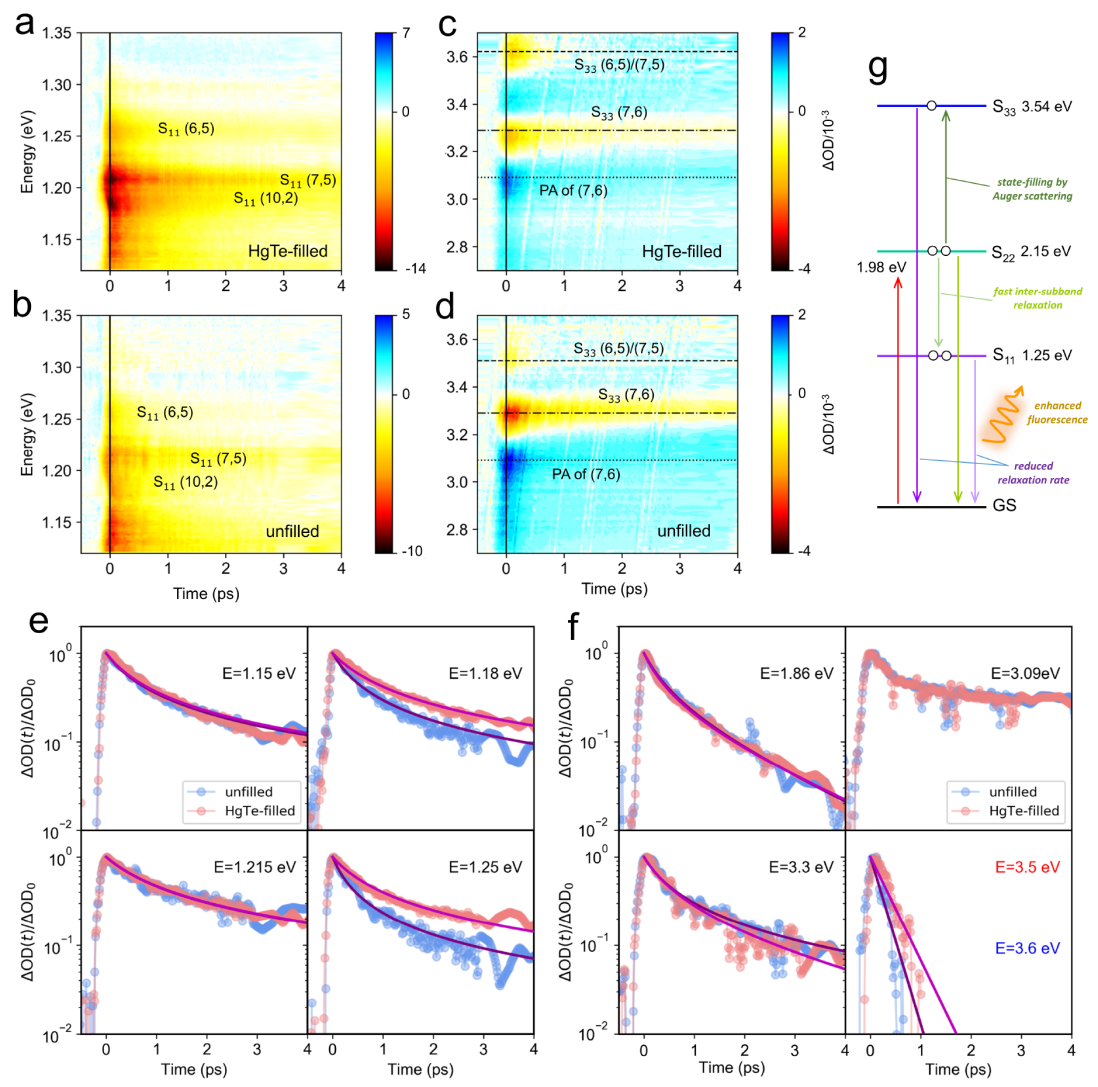
(a–d) Variations of the (a, b) S_11_ and (c, d) S_33_ transient absorption change (ΔOD) against pump–probe delay and probe energy for (a, c) HgTe-filled and (b, d) unfilled semiconducting SWCNTs in solution. The horizontal lines in panels (c) and (d) mark the energies of the different photobleaching (PB) and photoabsorption (PA) features. The pump photon energy and fluence were 1.98 eV (625 nm) and 1.1 mJ/cm^–2^ per pulse, respectively. (e, f) Normalized TA kinetics at different energies, (e) in the S_11_ spectral region and (f) in the S_*2*2_ and S_33_ regions. Fits (purple for the unfilled and magenta for the HgTe-filled) are as described in the text based on the bimolecular rate equation model in eq 3). This model did not reproduce the dynamics of the PA feature at around 3.09 eV. (g) Energy diagram of (6,5) and possible exciton relaxation pathways, under the influence of HgTe NW filling, which reduced the S_33_ and S_11_ Auger rates and thus enhanced its fluorescence. The upward red arrow denotes the pump.

Finally, we investigated the impact of HgTe zigzag filling on the transient absorption spectra and dynamics for semiconducting SWCNTs in solution, under excitation at 625 nm, corresponding to the S_22_ continuum for (7,5) and (7,6) tubes. This excitation condition was adopted so as to allow the dynamics of S_11_, S_22_, and S_33_ excitonic absorption features to be determined, as reported in Figure 5(a) and (b) (S_11_ resonances) and Figure 5(c) and (d) (near the S_33_ resonances). After the pump pulse depopulates the second highest valence band, rapid intersubband electron scattering within 50 fs results in a reduced electron density in the highest valence band.^56^ Thus, the excitonic S_11_ and S_22_ both exhibit ground-state bleaches with similar onset dynamics (<500 fs). Here we discuss the recovery dynamics.

The S_11_ spectral region was relatively congested, with ground-state bleach features associated with (10,2) SWCNTs at 1.18 eV, (7,5) at 1.21 eV, and (6,5) at 1.25 eV evident in Figure 5(a). The S_11_ energies for (7,6), (9,4), and (8,4) tubes were below the minimum energy accessible in the TA experiments (1.12 eV). Filling with HgTe chains appears to strengthen some of the ground-state bleach features, as well as slow some of the dynamics. This is evident in Figure 5(e), where transients at fixed energies are reported. At 1.15 and 1.215 eV, filling did not alter the dynamic, which could be modeled with γ*n*_0_ = 1.80 ± 0.02 THz and γ*n*_0_ = 1.13 ± 0.02 THz, respectively, ignoring *k*. While the dynamics at 1.215 eV corresponds to S_11_ of (7,5), the weaker TA signal at 1.15 eV (1078 nm) could result from the tail of the absorption of (10,2) or (9,4) tubes (Figure 2(e)). The TA signal at 1.18 eV becomes relatively stronger (Figure 5(a)) and slower (Figure 5(e)) after filling, and the Auger rate dropped from γ*n*_0_ = 2.37 ± 0.04 THz to γ*n*_0_ = 1.37 ± 0.01 THz with HgTe-encapsulated nanowires. A similar trend was found for (6,5), with γ*n*_0_ = 3.28 ± 0.05 THz, reducing to γ*n*_0_ = 1.50 ± 0.02 THz after filling. A lower Auger rate is consistent with an enhanced PL emission efficiency, as observed upon HgTe encapsulation for most chiralities (Figure 2(d) and (e)), since a greater fraction of excitons recombine radiatively when γ is reduced. Parameters for the fitted TA curves can be found in Table S3. An energy level diagram for HgTe-filled (6,5) SWCNTs is illustrated in Figure 5(g).

Turning to the kinetics of the higher excitonic transitions, the S_22_ resonances of (7,6) and (7,5) overlap in energy at around 1.93 eV and cannot be independently resolved. The combined S_22_ dynamics were probed at 1.86 eV, sufficiently far from the pump photon energy, and are reported in Figure 5(f) to be independent of filling. The S_22_ decay kinetics are somewhat faster than the S_11_ kinetics and were fit by a monomolecular lifetime τ = 1/*k* = 1.7 ps and γ*n*_0_ = 2.10 ± 0.07 THz. We interpret the bimolecular rate again as Auger two-particle recombination, while the monomolecular rate represents the time scale for S_22_ excitons to transfer to the S_11_ manifold *via* single-particle processes.

The ground-state bleach signals around 3.3 and 3.5–3.6 eV (Figure 5(c),(d)) can be assigned using the PLE results in Figure 2(d) and (e) to the (7,6) and (7,5) S_33_ resonances, respectively. The exact energies of the S_33_ and higher energy transitions have been discussed in depth in the literature,^57,58^ with the consensus emerging that rather than representing transitions to bound excitonic states, S_33_ transitions can be to unbound electron–hole pairs (*i*.*e*., the picture of transitions between single-particle van Hove singularities applies), as the exciton binding energy is small or negligible. Here, given the pump energy (1.98 eV) was below the energy region probed, the observed ground-state bleach absorption change can be understood as resulting from the depopulation of the ground state,^6^ as well as filling the upper state *via* Auger scattering, which may promote an S_22_ exciton into an S_33_ state.^59^ While the S_33_ (7,6) dynamics were not affected by HgTe encapsulation (Figure 5(f)), the transient absorption for (7,5) was blue-shifted from 3.5 eV to 3.6 eV after filling, and the decay dynamics were slowed from a monomolecular lifetime τ = 0.18 ps to 0.31 ps after filling (fit parameters are reported in Table S4). Note that the (7,5) S_11_ dynamics were not altered by filling.

These results imply that HgTe filling may alter the higher lying electronic states in a complex manner that depends on the chirality of the SWCNT. One mechanism for this is that a zigzag chain should distort the encapsulating SWCNT’s cross-section into an oval,^17^ modifying the electronic wave function. Moreover, a positive photoabsorption (PA) feature positioning at around 3.09 eV was discovered, with distinctly slower recombination than for the (7,5) and (7,6) S_33_ features. The dynamics at 3.09 eV were also not modified after filling with the HgTe NWs (Figure 5(f)), and the decay could not be fit well by the bimolecular model. We speculate that this PA feature may represent a transient bandgap renormalization or a change in the line width of the (7,6) S_33_ resonance.

## Conclusion

In summary, we demonstrated experimentally that encapsulated HgTe NWs adopt a zigzag structure inside sub-1-nm-wide SWCNTs. Semiconducting SWCNTs (a few microns long) and metallic SWCNTs (less than 200 nm long) were successfully extracted from the as-prepared HgTe NW-filled raw product by a gel column chromatography method. Electron microscopic imaging revealed a dominance of zigzag atomic chains in both the semiconducting and metallic SWCNTs. Steady-state optical characterization, including photoluminescence excitation spectroscopy and Raman spectroscopy, revealed that metallic and semiconducting (*n*,*m*) chiralities were altered differently by the HgTe NWs. In particular, the fluorescence of some semiconducting chiral (*n*,*m*) SWCNTs (*e*.*g*., (7,5) and (9,4)) was enhanced, while that from others (*e.g*., (7,6)) was significantly suppressed after the HgTe NW filling. The high-frequency Raman modes for the semiconducting and non-armchair (*n*,*m*) species displayed a downshifting after the HgTe filling, indicating an electron transfer from HgTe NWs to the SWCNTs and further suggested that filling enhanced electron–phonon coupling for the metallic SWCNTs. The impact of the NWs on electron–phonon and phonon–phonon coupling was investigated in the time domain *via* studies of the ultrafast transient absorption features of excitons, exciton–phonon side bands, and free carrier transitions. The faster Auger and phonon decay processes in metallic tubes after HgTe filling confirm theoretical predictions that encapsulated nanowires can modify the important functional properties of SWCNTs.

## Methods

### Materials

SWCNTs produced by the CoMoCAT (ref no. 775533) were used as raw carbon nanotube products. Sodium dodecyl sulfate (SDS, ACS reagent, ≥99.0%, Sigma-Aldrich) was used as the surfactant to isolate carbon nanotubes. Mercury telluride (HgTe, 99%, Alfa Aesar) was used as the filling material. Hydrogels made from a cross-linked copolymer of allyldextran and *N*,*N*′-methylene bis(acrylamide) (Sephacryl S-200, GE Healthcare) were applied for the gel column chromatography experiment.

### Nanowire Synthesis

Filling of SWCNTs by HgTe was conducted by melting infiltration.^12^ In brief, the raw powder of SWCNTs was mechanically mixed with HgTe powder (VMR 99.999%) and then loaded into the silica quartz ampule. After being sealed under vacuum, the ampule was heated to 770 °C for 12 h followed by 670 °C for 3 h. After heating, the HgTe-filled SWCNT powder was dispersed, centrifuged, and sorted based on the methods introduced below.

### SWCNT Dispersing and Sorting

The powder-like SWCNT raw material was added to an aqueous SDS solution (2 wt %) and then dispersed by a tip sonicator (150 W, 20% power output) under pulsed mode (2 s power on and 2 s power off) for at least 15 h. The catalyst particles and large nanotube bundles were removed by centrifugation at 197000*g* for 0.5 h. With this process, the sedimentation of SWCNTs was efficiently avoided.

The as-centrifuged SWCNT solutions were sorted based on a gel column chromatography approach similar to the one reported by a previous work,^60^ as distinct from the pure density gradient sorting approach.^61^ In brief, alkyl dextran-based gels were loaded into a glass column and equilibrated by a 2 wt % SDS solution. An SWCNT solution of about twice the volume of the gel bed was then applied to the column. After the SWCNT solution passed through the gel bed and a fraction of nanotubes were trapped, aqueous solutions containing increasing amounts of SDS (*e*.*g*., 0.3, 0.5, 1.0, 1.5, 2.0, 5.0 wt %) were used to elute the nanotubes. It has been found that the fraction of semiconducting nanotubes that have the smallest diameters (*e*.*g*., (6,4) and (6,5)) can be collected first. To sort nanotubes with greater diameters, pure DI water was added to the solution of SWCNTs that were not bound to the gels (to decrease the surfactant concentration to a certain value, such as 1.6 or 1.2 wt %), and the gel chromatography experiment was repeated. The outcome of diameter selection by diluting the SWCNT solution was evidenced in Figure S12, where a higher fraction of larger-diameter SWCNTs can be obtained with the concentration of SDS decreased from 2 wt % to 1.5 wt %. To extract metallic nanotubes from CoMoCAT76 SWCNTs, the as-centrifuged SWCNT solution was applied to the gel column and the first solution portion coming out from it (showing a light brown color) was collected, which had an enrichment of metallic SWCNTs.

### STEM Imaging

The nanostructure of the material was determined by a doubly corrected JEOL ARM200F TEM operated under the scanning mode. The microscope was equipped with CEOS imaging aberration, probe correction and a Gatan SC1000 ORIUS camera with a 4008 × 2672 pixel CCD. Imaging was conducted under an acceleration voltage of 80 and 200 kV, both of which were verified not to cause serious damage to the carbon structure. The SWCNT samples were loaded onto the lacey TEM grid by drop-casting. The TEM grids were baked in a vacuum oven at 100–150 °C for 12 h prior to characterization in order to remove contaminants such as hydrocarbon molecules. Image analyses were carried out in the Gatan Microscopy Suite (GMS) software.

### Chemical Composition Analysis

The chemical compositions of the SWCNT samples were studied by EDX characterization based on a Zeiss Gemini scanning electron microscope (SEM) equipped with a silicon drift detector. A high acceleration voltage of 20 kV and a sufficiently long working distance of 8.5 cm were applied in order to collect enough X-ray signals.

### AFM Imaging

The sample height and morphology of the material was studied by a Bruker Dimension Icon atomic force microscope. Measurements were carried out under peak force tapping mode at a tapping rate of 2 kHz, with an aluminum-coated SiN cantilever (tip radius: 2–12 nm, spring constant: 40 N/m, resonant frequency: ∼70 kHz, length: ∼115 μm, width: ∼25 μm) applied as the probe. While scanning, the instrument also collects the error signal (the difference between set point amplitude value and actual amplitude value), which provides the information on interaction between the cantilever tip and sample surface. The set point force value was kept under 1 nN (0.5–1 nN) throughout the experiment. The plane fit method was used to flatten (improve the quality of) the as-obtained image.

### Absorbance Spectroscopy

The equilibrium-state absorbance of SWCNT samples was characterized by a Cary60 instrument (detecting 190–1100 nm or 6.53–1.13 eV), a PerkinElmer Lambda 1050 UV–vis–NIR spectrometer (260–1300 nm or 4.77–0.95 eV), and a Bruker Vertex 70 V Fourier-transform infrared spectrometer (130–2500 cm^–1^ or 0.016–0.31 eV). Both thin-film SWCNT samples, produced by a vacuum filtration approach, and solution-state samples were examined. For UV–vis–NIR absorbance measurement on solution samples, background signals attributed to the surfactant medium were subtracted. For the mid- and far-IR measurements performed on the FTIR spectrometer, a solid-state silicon beam splitter was used.

### Fluorescence Spectroscopy

The photoluminescence of SWCNT solutions was examined by a Horiba Fluorolog-3 spectrometer equipped with a xenon lamp that generated a broadband white light beam. A single-grating monochromator was applied to select the excitation wavelength. A photomultiplier tube and a liquid nitrogen-cooled InGaAs detector were used to detect the fluorescence signals in the UV–vis range (below 850 nm) and at near-infrared (850–1350 nm) wavelengths, respectively. The spectrometer corrected for variations in the lamp output (by counting excitation signals from a silicon photodiode detector) and the detector monochromator’s sensitivity. Before the measurement, the SWCNT liquid dispersion was loaded into a cuvette with 1 cm path length. The fluorescence was collected in a right-angle geometry. To select the excitation light in S_22_ and S_33_ wavelengths, a 490 nm long-pass filter and a 330–660 nm band-pass filter were placed after the excitation grating slit, respectively. An NIR long-pass glass filter was placed before the emission grating slit to block Rayleigh scattered light. To characterize the phonon sidebands of the SWCNTs, measurements were carried out at excitation wavelengths between 850 and 1000 nm, which required changing the excitation grating from 1200 lines/mm to 600 lines/mm.

### Raman Spectroscopy

Raman spectra were investigated using either Renishaw InVia Reflex or Horiba LabRam HR Evolution spectrometers. The LabRam spectrometer was equipped with a laser excitation of 600 or 488 nm, with both lasers providing a maximum optical power of 50 mW at the sample and a 600 l/mm grating. The inVia systems were equipped with 442 nm (25 mW), 514.5 nm (38 mW), 532 nm (20 mW), and 633 nm (10 mW) with maximum powers at the sample as indicated. All data on the Invia system were collected using a 1800 l/mm grating except for the 442 nm excitation, for which a 2400 l/mm grating was used. To get a desired S/N ratio while avoiding heating or damaging the sample, the laser power was reduced to 1% to 5% of its maximum, except for the case of 442 nm excitation, for which a power output of 50% was applied to get a sufficient signal intensity. The laser beam was brought to a micron-scale spot focus (<5 μm) onto the sample by a 50× objective lens, with an NA of 0.9. Spectra were collected in the backscattering configuration. The samples for Raman measurement were prepared by a drop-casting method on a quartz substrate.

### Transient Absorption Spectroscopy

The exciton dynamics of solution-state SWCNTs were examined using a TA spectrometer. Both the pump beam and the probe beam were derived from an optical parametric amplifier (TOPAS), which was seeded with a 1 kHz, 40 fs, 800 nm pulse generated by an amplified Ti:sapphire laser (Newport Spectra Physics Spitfire Ace PA). The pump beam was mechanically chopped at 500 Hz. Different white light probe continua (330–720 and 700–1100 nm) were produced from a CaF_2_ crystal pumped at 800 nm and a sapphire crystal pumped at 1300 nm, respectively. A set of neutral-density filters and narrow band-pass filters were placed in the beam path to avoid saturation of the detector while affording a broadband white light supercontinuum. The pulse width/duration of the setup was 40 fs, which defined the resolution of the experiment. The acquired TA signals were chirp-corrected. For the semiconducting SWCNT samples, a pump wavelength of either 625 or 690 nm was chosen to satisfy the condition of S_22_ resonance. For the metallic SWCNT samples, a pump wavelength of 440 nm, which was slightly shorter than the M_11_ wavelengths (∼460 and ∼507 nm), was chosen to avoid pump scatter in the probe beam direction.

## Supporting Information Available

Additional microscopy images; EDX, UV–visible, PL, and Raman spectra; fit parameters (PDF)
