# Supplementary material for: Zigzag HgTe Nanowires Modify the Electron–Phonon Interaction in Chirality-Refined Single-Walled Carbon Nanotubes
Source: ACS Nano. 2022 Apr 7;16(4):6789–800. doi: 10.1021/acsnano.2c01647 (PMC9046977; doi:10.1021/acsnano.2c01647)
Supplement: Supplementary file 1 — nn2c01647_si_001.pdf [file nn2c01647_si_001.pdf]

Supplemental Information for: Zig-zag HgTe nanowires modify the electron-phonon interaction in chirality-refined single-walled carbon nanotubes

Ziyi Hu,* Ben Breeze, Reza J. Kashtiban, Jeremy Sloan,* and James Lloyd-Hughes*

University of Warwick, Department of Physics, Gibbet Hill Road, Coventry, CV4 7AL, United Kingdom.

E-mail: ziyi.hu@warwick.ac.uk; j.sloan@warwick.ac.uk; j.lloyd-hughes@warwick.ac.uk

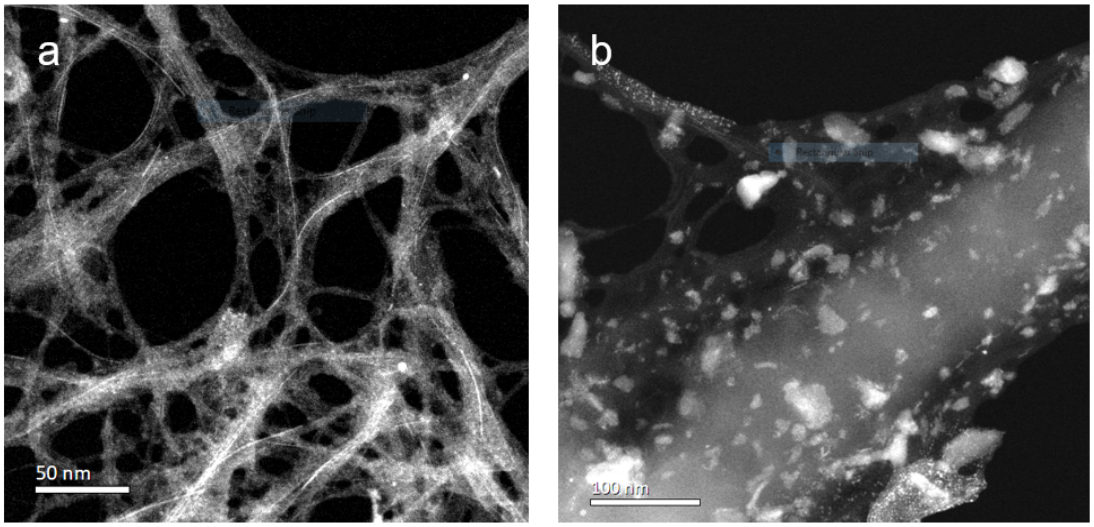

Figure S1. ADF-STEM images of the HgTe-filled SWCNT samples after centrifugation at (a) 197000×g and (b) 5000×g.

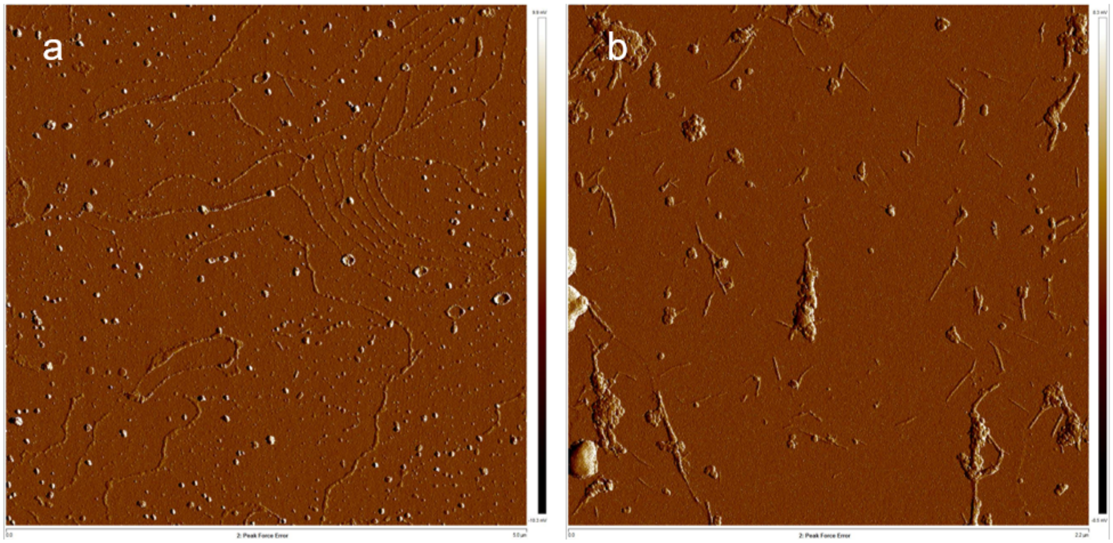

Figure S2. Peak-force error maps for (a) semiconducting and (b) metallic SWCNT samples.

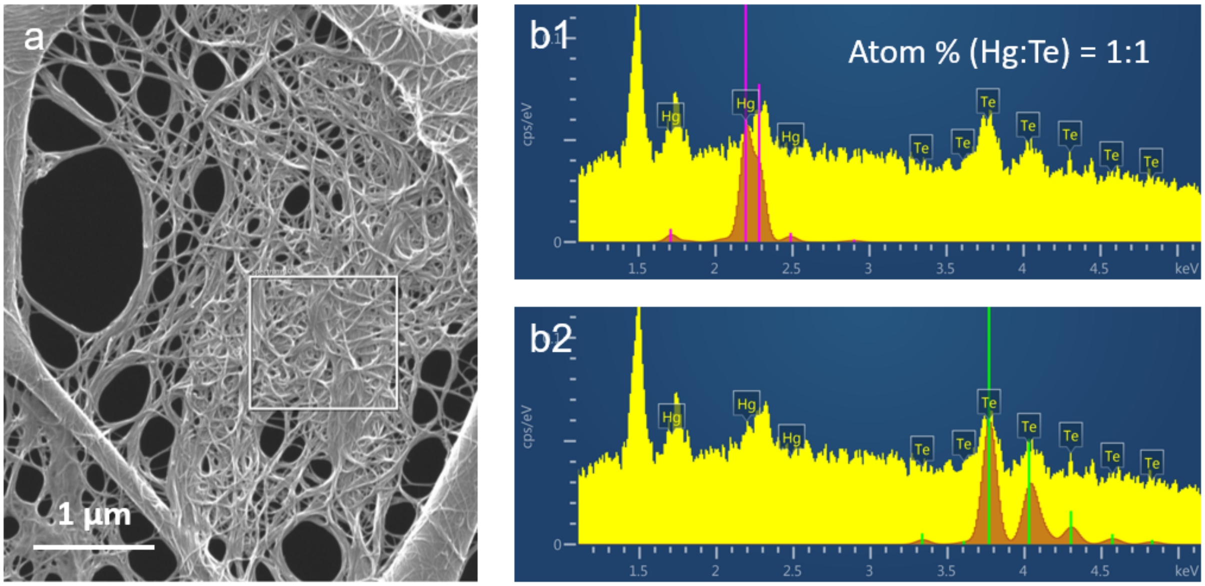

Figure S3. EDX analysis on the HgTe-filled SWCNT sample. (a) The morphology of SWCNT film in the scanned region. The white rectangle highlights the area selected for EDX detection. (b) The experimental (yellow spectral region) and simulated (red spectral regions) EDX signal peaks.

S20.PNG

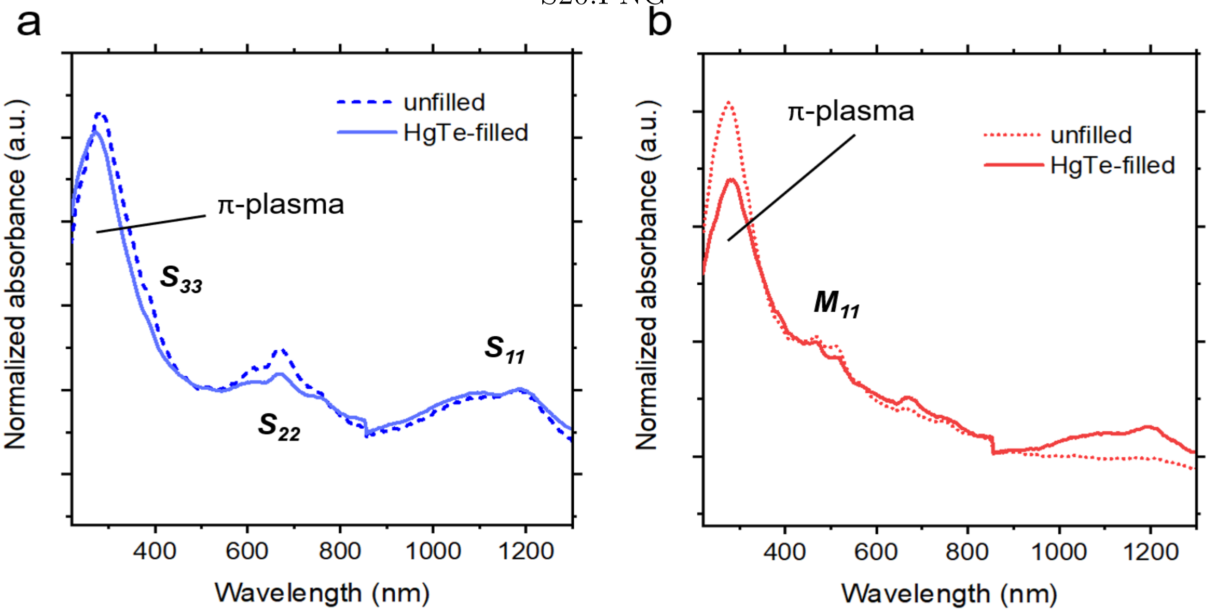

Figure S4. UV-Vis-NIR absorbance spectra of the thin film-state (a) semiconducting SWCNTs and (b) metallic SWCNTs produced by vacuum filtration. The spectra for the semiconducting and metallic SWCNTs were normalized to the absorbance values at 536 nm and 440 nm, respectively, where there were no expected excitonic features. The sharp peaks presenting at ∼265-280 nm were assigned to the component of π-plasma. The artifacts at ∼860 nm were induced by the change of detector during the scan.

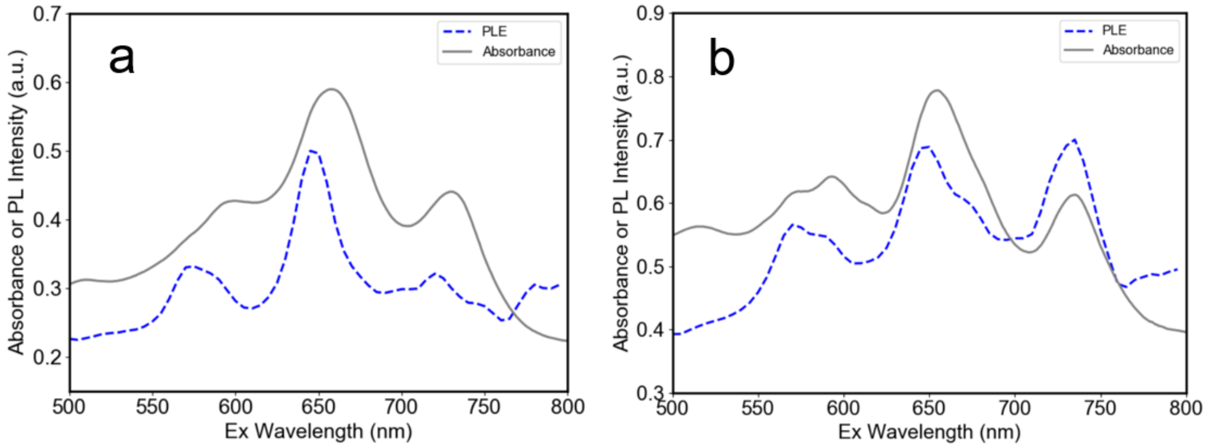

Figure S5. Absorbance spectrum and PLE averaged over the entire spectral window (850-1350 nm) of the (a) unfilled and (b) HgTe-filled semiconducting SWCNT sample.

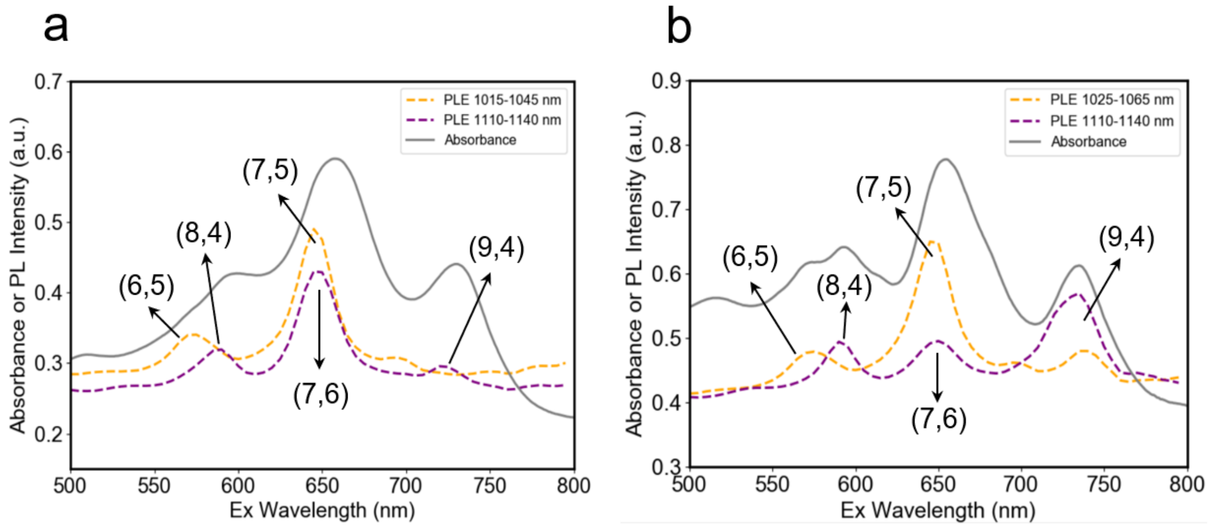

Figure S6. Absorbance spectrum and PLE averaged over two specific spectral windows of the (a) unfilled and (b) HgTe-filled semiconducting SWCNT sample. The orange and purple PLE profiles represent contributions of emission signals from (6,5)/(7,5) and (8,4)/(7,6)/(9,4), respectively.

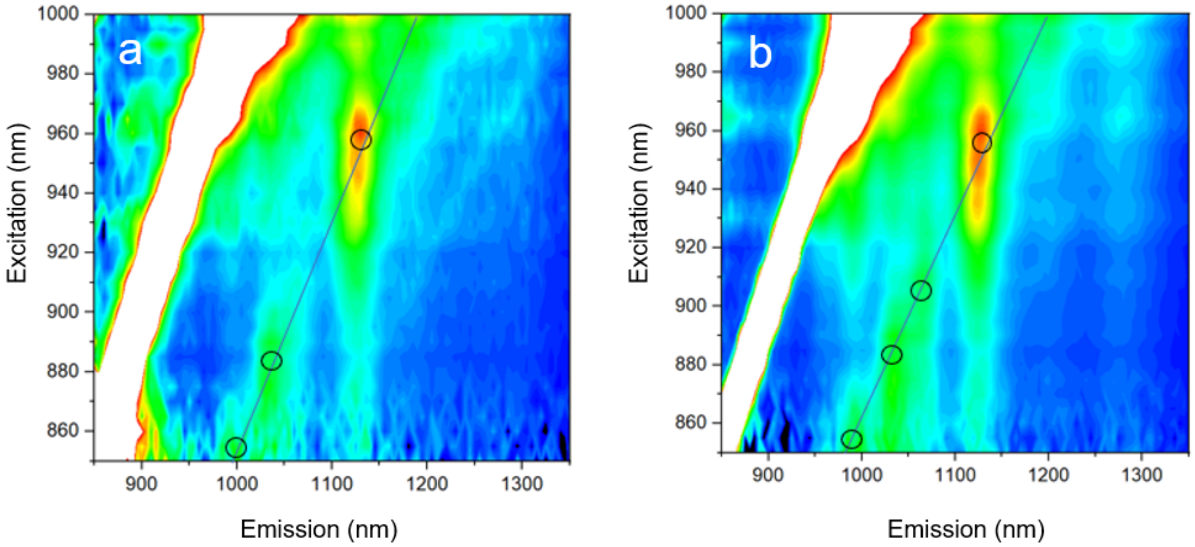

Figure S7. PLE maps of the unfilled and HgTe-filled semiconducting SWCNT samples by excitation in the NIR wavelengths. The phonon sidebands for S_{11} excitons of different (n, m) are marked by black open cycles.

Table S1. Fitting parameters for the BWF lines of unfilled and HgTe-filled metallic SWCNTs

	ω_{BWF} (cm^{-1})	$1/q$	Γ (cm^{-1})
488 nm excitation			
unfilled	1547	-0.22	25
HgTe-filled	1543	-0.45	31
514 nm excitation			
unfilled	1550	-0.16	21.5
HgTe-filled	1546	-0.23	26
532 nm excitation			
unfilled	1552	-0.13	22.9
HgTe-filled	1547	-0.18	28

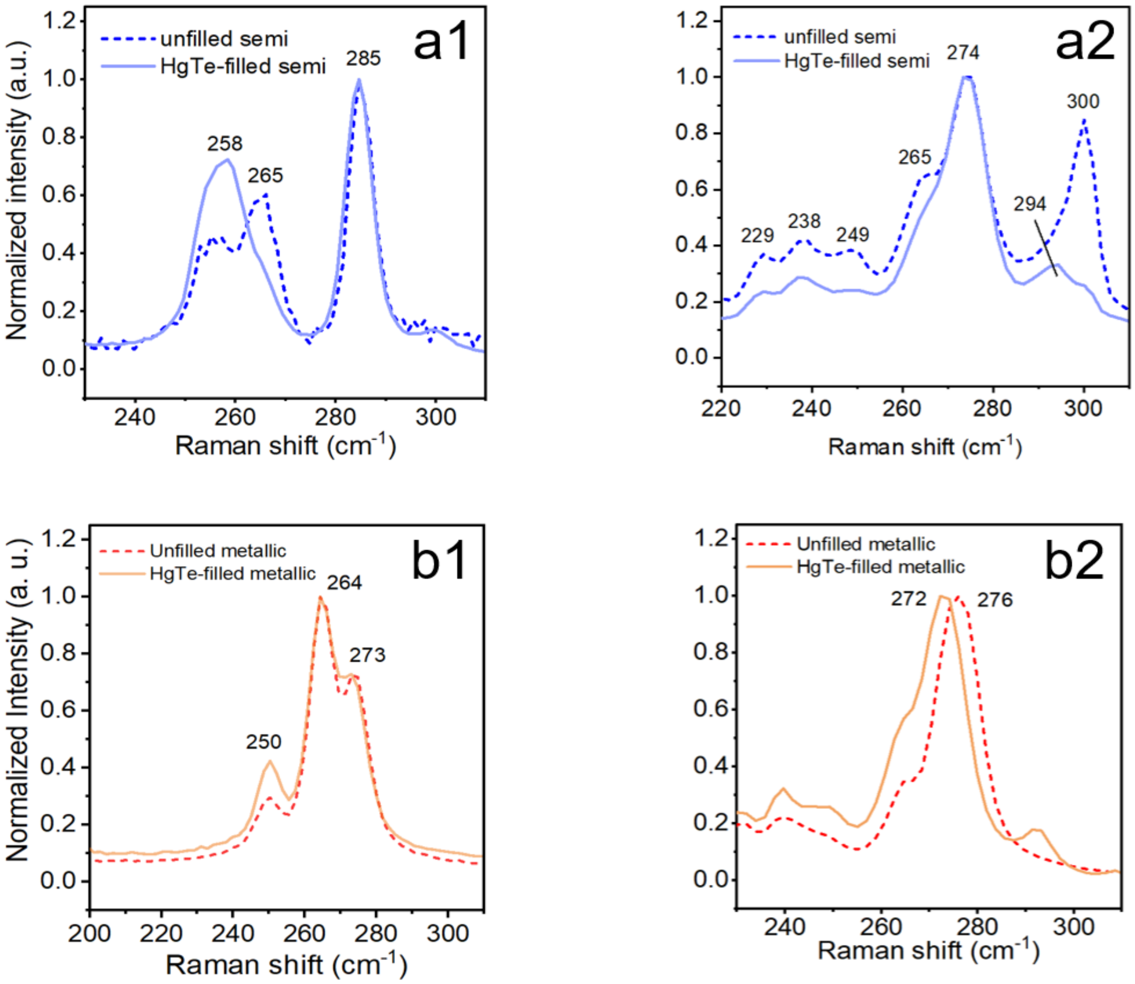

Figure S8. Raman RBM spectra of (a1,a2) semiconducting SWCNT samples under 633 nm and 532 nm excitation and (b1,b2) metallic SWCNT samples under 514 nm and 532 nm excitation. The signal peak centered at 273 cm^{-1} is assigned to (8,4).

Table S2. Fitting parameters for the M_{11} TA kinetics of unfilled and HgTe-filled metallic SWCNTs

	2.695 eV (M_{11} of (6,6))	2.445 eV (M_{11} of (8,5))	2.231 eV (G mode of (8,5))
γn_0 (THz)			
unfilled	3.27 ± 0.09	4.13 ± 0.06	4.13 ± 0.21
HgTe-filled	5.07 ± 0.20	7.17 ± 0.13	-
k (THz)			
unfilled	-	-	1
HgTe-filled	-	-	6.25

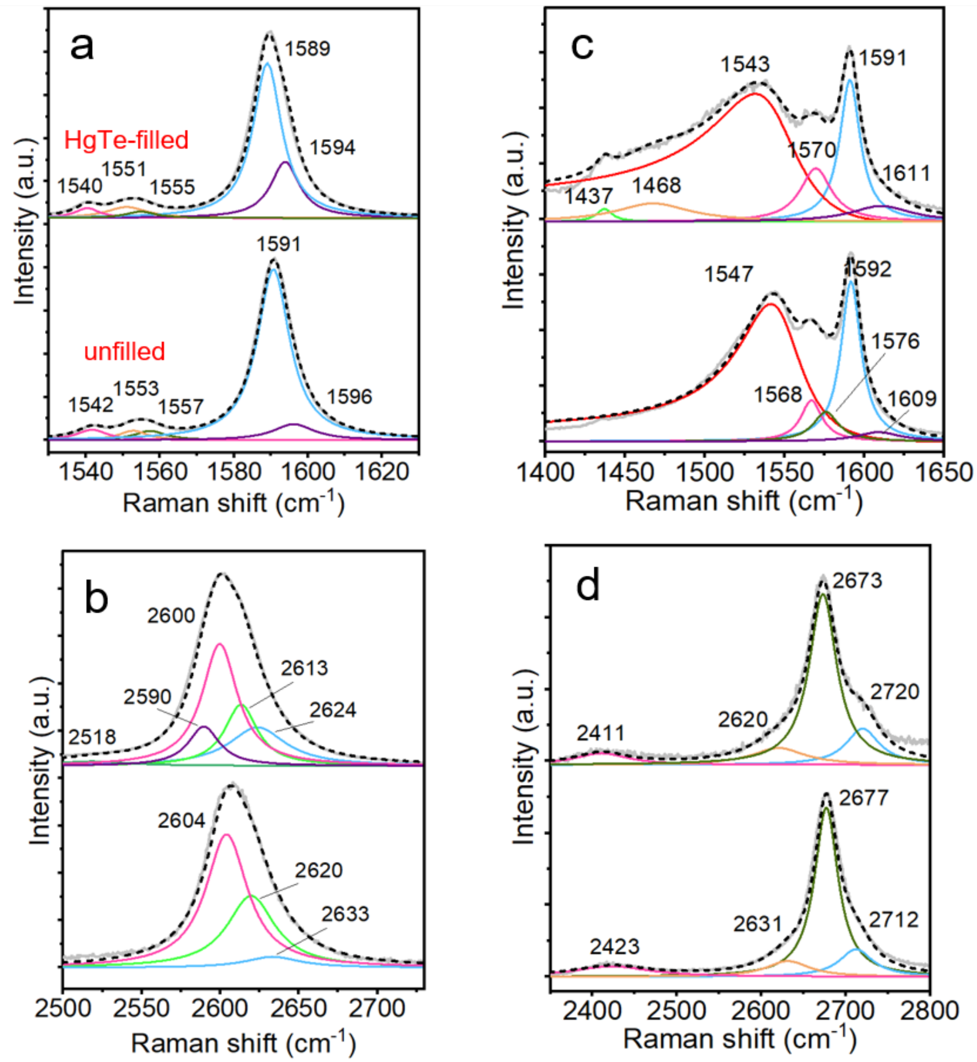

Figure S9. High-frequency G and G' modes of (a,b) semiconducting SWCNTs under 633 nm excitation and (c,d) metallic SWCNTs under 488 nm excitation.

Table S3. Fitting parameters for the S_{11} TA kinetics of unfilled and HgTe-filled semiconducting SWCNTs. No k term was needed.

	1.15 eV	1.18 eV	1.215 eV	1.25 eV
γn_0 (THz)				
unfilled	1.84 ± 0.02	2.37 ± 0.04	1.14 ± 0.02	3.28 ± 0.05
HgTe-filled	1.73 ± 0.03	1.37 ± 0.01	1.12 ± 0.01	1.50 ± 0.02

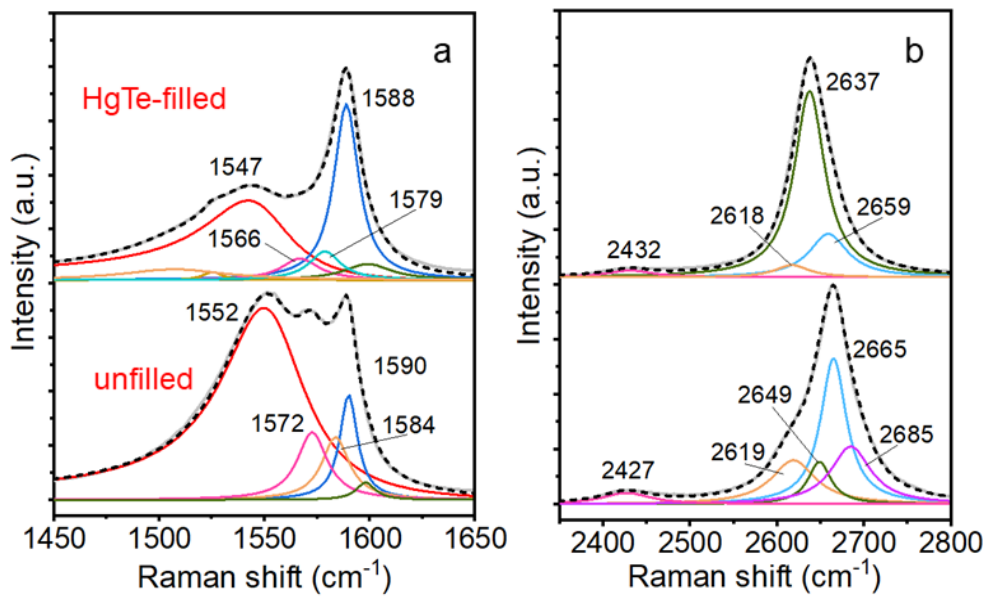

Figure S10. G and G' modes of the metallic SWCNT samples under 532 nm excitation.

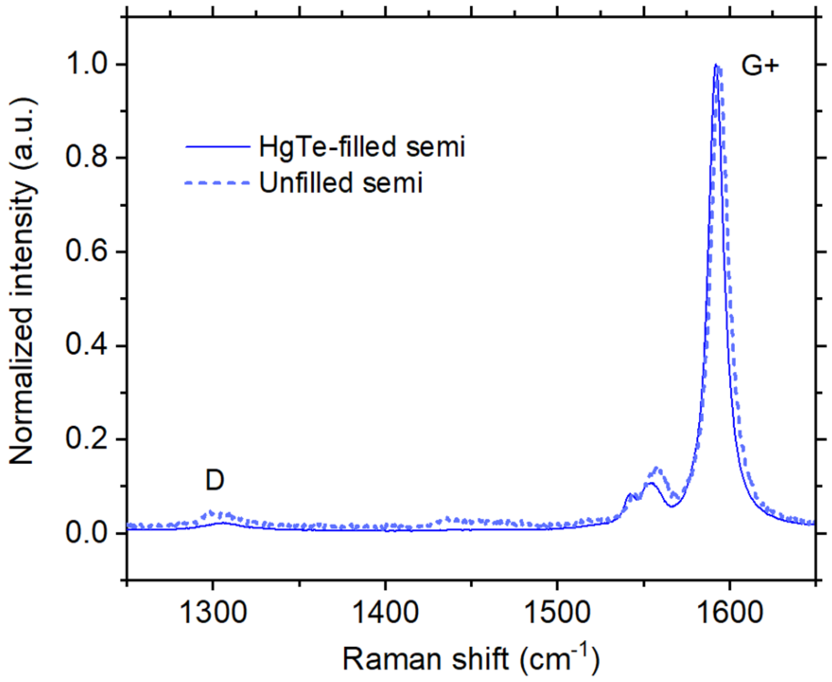

Figure S11. G and D modes of the HgTe-filled semiconducting SWCNT sample under 660 nm excitation.

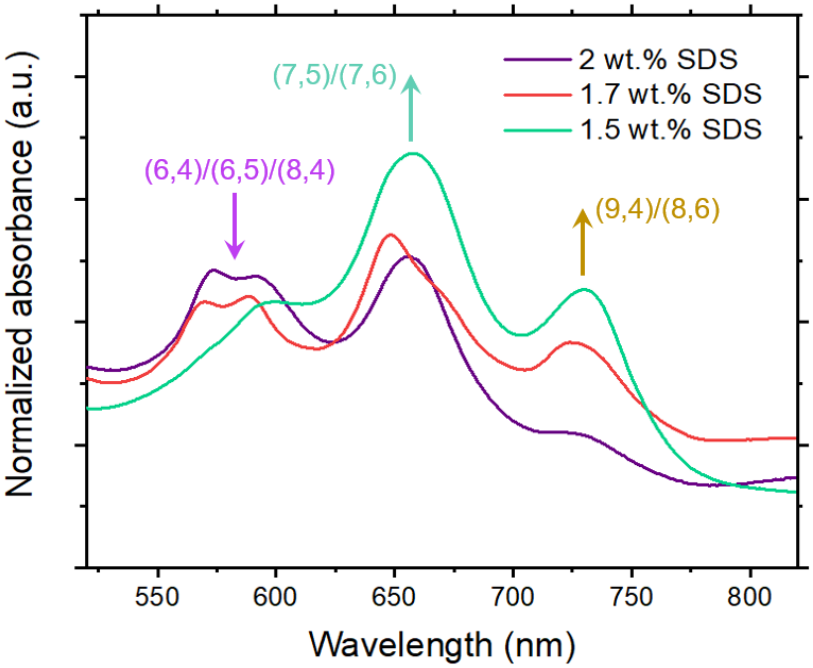

Figure S12. UV-Vis-NIR absorption spectra of the unfilled semiconducting SWCNT solutions sorted from the SWCNT dispersions that had different concentrations of SDS. In all cases, the sorted nanotubes were fractions eluted by 5 wt. % SDS aqureous solutions. The spectra were normalized to their absorbance values at 400 nm. Changes in the absorbance attributed to the S_{22} transitions as a result of decrease in the SDS concentration indicated that larger-diameter SWCNTs were prone to be selected at lower SDS concentrations.

Table S4. Fitting parameters for the S_{22} and S_{33} TA kinetics of unfilled and HgTe-filled semiconducting SWCNTs

	1.86 eV	3.3 eV	3.5 eV (unfilled)	3.6 eV (HgTe-filled)
γn_0 (THz)				
unfilled	2.00 ± 0.06	2.55 ± 0.06	-	-
HgTe-filled	2.27 ± 0.07	2.4 ± 0.1	-	-
k (THz)				
unfilled	0.62	-	0.56	-
HgTe-filled	0.57	0.21	-	0.32
